# 4D Clinical Imaging for Dynamic CAD

**DOI:** 10.1155/2013/690265

**Published:** 2013-09-04

**Authors:** Mark Lauren, Frederick McIntyre

**Affiliations:** ^1^4D Dental Systems, Inc., 106 Westfield Road, Amherst, NY 14226, USA; ^2^American Board of Prosthodontics, P.O. Box 665, Amherst, MA 14226, USA

## Abstract

A basic 4D imaging system to capture the jaw motion has been developed that produces high resolution 3D surface data. Fluorescent microspheres are brushed onto the areas of the upper and the lower arches to be imaged, producing a high-contrast random optical pattern. A hand-held imaging device operated at about 10 cm from the mouth captures time-based perspective images of the fluorescent areas. Each set of images, containing both upper and the lower arch data, is converted to a 3d point mesh using photogrammetry, thereby providing an instantaneous relative jaw position. Eight 3d positions per second are captured. Using one of the 3d frames as a reference, incremental transforms are derived to express the free body motion of the mandible. Conventional 3d models of the dentition are directly registered to the reference frame, allowing them to be animated using the derived transforms.

## 1. Introduction

The incorporation of patient-specific motion into the CAD used for dental prosthetics remains a desirable goal. Beginning with the work of Karlsson [1], photogrammetry has been increasingly applied to the study of the mandibular motion. Photogrammetry targets have been applied to frames [2–4] and shown capable of characterizing the mandibular motion. Targets have also been attached to the teeth using a variety of methods [5–9]. An inherent problem with the use of frames and individual targets is the required registration of the dynamic clinical data to the 3D anatomy of interest. Each reported method requires a specific technique to achieve this registration.

This report presents a brief description of a new mandibular imaging system, the derivation of an associated 4D model, and its integration into CAD. The present system essentially takes expanded buccal scans at 8 Hz with sufficient data to define a local relative jaw position. Since 3D surface files are produced, they can be used to (1) derive incremental transforms to characterize the motion and (2) directly register the 3D oral anatomy of interest to be animated.

## 2. Imaging Method

Fluorescent polystyrene microspheres (beads) of about 20 *μ*m diameter are brushed onto the areas of the upper and the lower arches to be imaged, spanning about six teeth. These areas should lie within the oral anatomy of interest to be animated. Both hard and soft tissues can be imaged. The purpose of the beads is to produce a random high-contrast optical pattern to the imaging system. 

**Figure 1:**
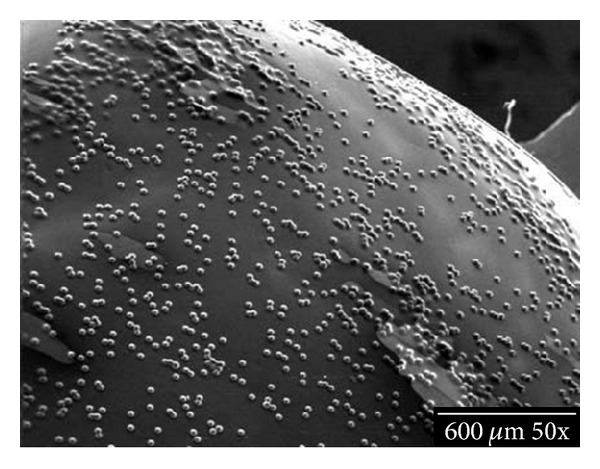
A low power SEM shows the random distribution of the beads on a tooth. Open areas without beads contribute to the random field and provide good imaging.

**Figure 2:**
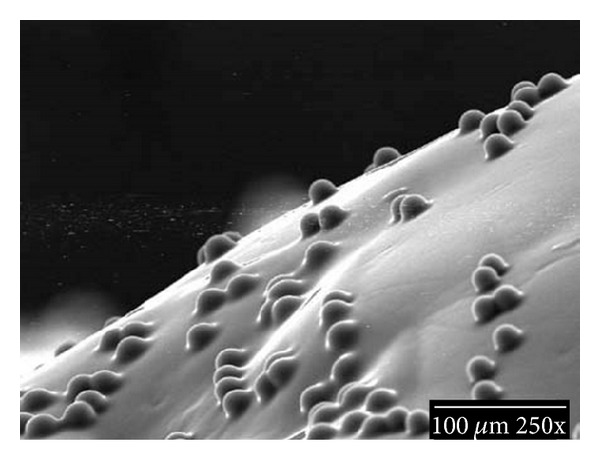
A medium power SEM shows 25 *μ*m diameter beads covered by a thin polymer film.

The beads are suspended in a viscous ethanol solution containing polyvinyl pyrrolidone and a colorant. After brushing on, the alcohol evaporates leaving the microspheres trapped under a thin polymer film. The polymer film is water soluble, and the film/bead material is nontoxic. See Figures 1 and 2. 

**Figure 3:**
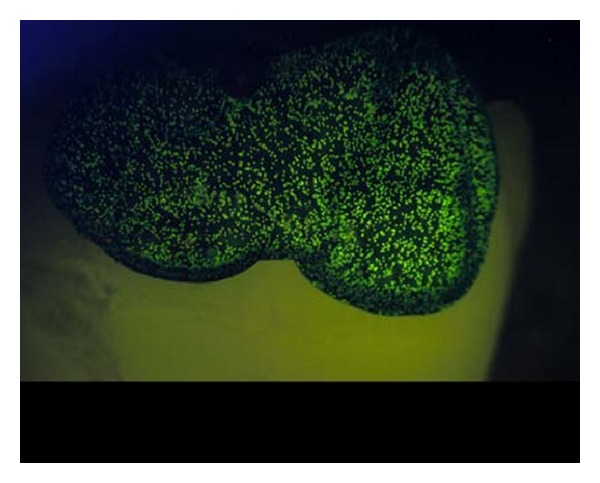
A color image of a bead film taken under blue light and a green bandpass filter. The film provides a dark background allowing direct imaging over the fluorescing enamel.

The beads fluoresce green under the blue light from the imaging unit.  Figure 3 is a colored image taken under the blue LED illumination and through a green bandpass filter. This filter allows only the fluorescing wavelength (500–600 nm) to pass and be imaged. The incident blue light also causes the tooth enamel to fluoresce, but the colored film blocks the fluorescence coming from the underlying enamel, rendering the background dark. This produces the desired high-contrast field for 3D imaging. 

## 3. Imaging Device

**Figure 4:**
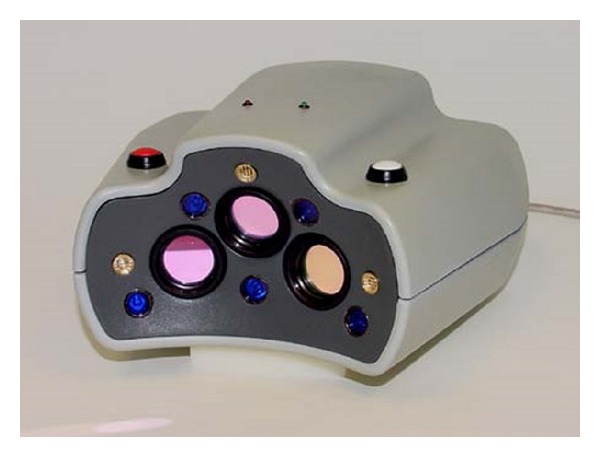
The hand-held imaging unit is about 6 inches wide and 8 inches deep. The white button activates the blue lights and red lasers, both of which are blocked by the green bandpass filter and are not imaged. The red button is for recording.

The imaging unit shown in Figure 4 contains three cameras mounted at low angles and five blue LEDs on the front face. Monochrome cameras are used for clinical imaging, which show the fluorescing beads as white against a dark background. Three laser diodes (gold discs) project red alignment beams to assist the user with positioning the device. Two crossing vertical lines are used for distancing and tilting, while a horizontal line is positioned along the occlusal plane. At the 10 cm working distance, the unit has a field of view 53 × 42 mm and a 40 mm depth of field. 

The three cameras fire simultaneously, eight times per second, producing 24 images per second. The 3D surface of the applied patches is derived from each set of three images. A typical imaging sequence lasts 12 seconds. Over this time, ninety-six 3D positions are captured. A single USB line is used for power and data transfer. 

## 4. Clinical Imaging

Clinical imaging is relatively straightforward, since the teeth do not require special preparation. The basic steps include

1. using cheek retractor for access,
2. brushing on bead patches using a nylon or a microbrush; Film dries in 10 sec,
3. imaging,
4. rinsing with warm water to remove film.

The 8-bit monochrome camera images are then transmitted to the laboratory for processing.

## 5. Deriving 3D Surfaces

**Figure 5:**
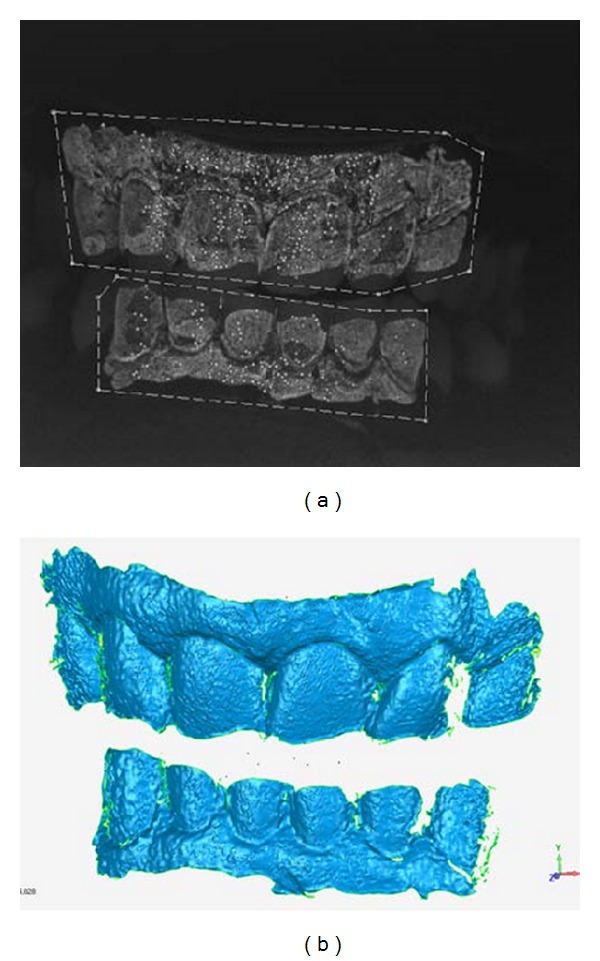
An example of a monochrome camera image and the associated 3D surface file. The upper and lower fluorescent regions are readily seen as well as the exposed fluorescing enamel. The white dots are part of the image alignment process used by the PhotoModeler software, and the two surrounding perimeter lines define the separate fields to be analyzed.

The PhotoModeler software from EOS Systems (Vancouver, CA) is used. For each set of three images, two 3D point meshes are first derived: one using the center and left side camera images, and one using the center and right side camera images. These two-point meshes are then merged into a single mesh at a controlled point spacing and saved as a 3D file in standard formats.  Figure 5 shows a single camera image and the corresponding 3D surface. A set of 3D files of the fluorescent areas is produced for each clinically captured motion sequence. The point-to-point accuracy of the system is about 10 *μ*m.

## 6. 4D Model

**Figure 6:**
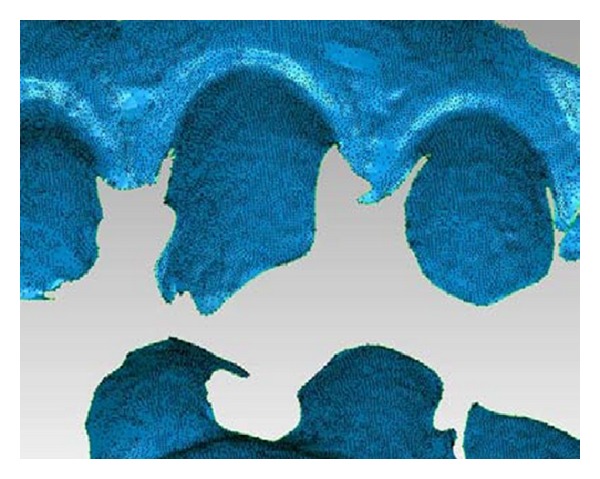
A typical 3D camera frame at 80 *μ*m point spacing.

**Figure 7:**
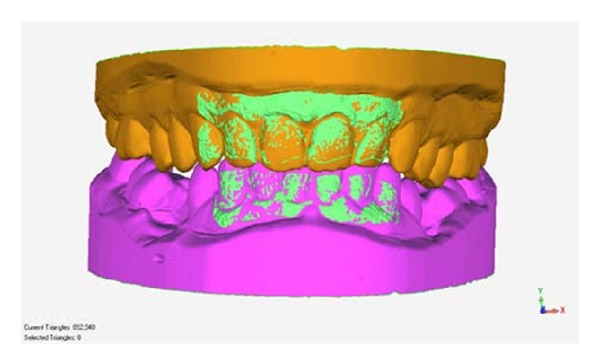
The registration of complete upper and lower 3D models to the camera frame in Figure 5. The production of 3D files allows the oral anatomy of interest to be directly registered to the camera data.

The time-based 3D frames are used to derive a corresponding set of six degree-of-freedom (dof) expressions to describe the mandible's free body jaw motion. First, one of the frames in a sequence is defined as a reference. Any frame can be used for this purpose. This frame defines the fixed upper position and provides a lower reference position. The upper data in frame *n* is then registered to the upper reference position. The lower data in frame *n* is now displaced from the lower reference position. Then, the lower reference position is registered to the lower data from frame *n*. This provides the dof expression (transform) for frame *n*, consisting of three translation and three rotation terms. In this way, a dof expression is derived for each frame to define the mandible's incremental free body motion for a particular clinical sequence. The incremental transforms are then used to drive animations. The large amount of curvature associated with these files helps provide accurate registrations (see Figures 6 and 7). 

## 7. Animations

The first step in animation is to register the extended anatomy of interest to the reference frame of a sequence. This anatomy is obtained either by intraoral scanning or scanning stone models. 

Simulations are generally run at 24 frames/sec. Since the camera system produces data at 8 Hz, Maya 3D animation software from Autodesk, Inc. (San Rafael, California, USA) is used to interpolate two intermediate positions between each camera frame to produce a smoother animation. Each position is then saved as a 3D  pdf file. Animations are then run using Adobe Acrobat X Pro (Adobe Systems Incorporated, San Jose, California, USA). Similarly, driven animations can be performed in CAD to display and analyze the motion. 

**Figure 8:**
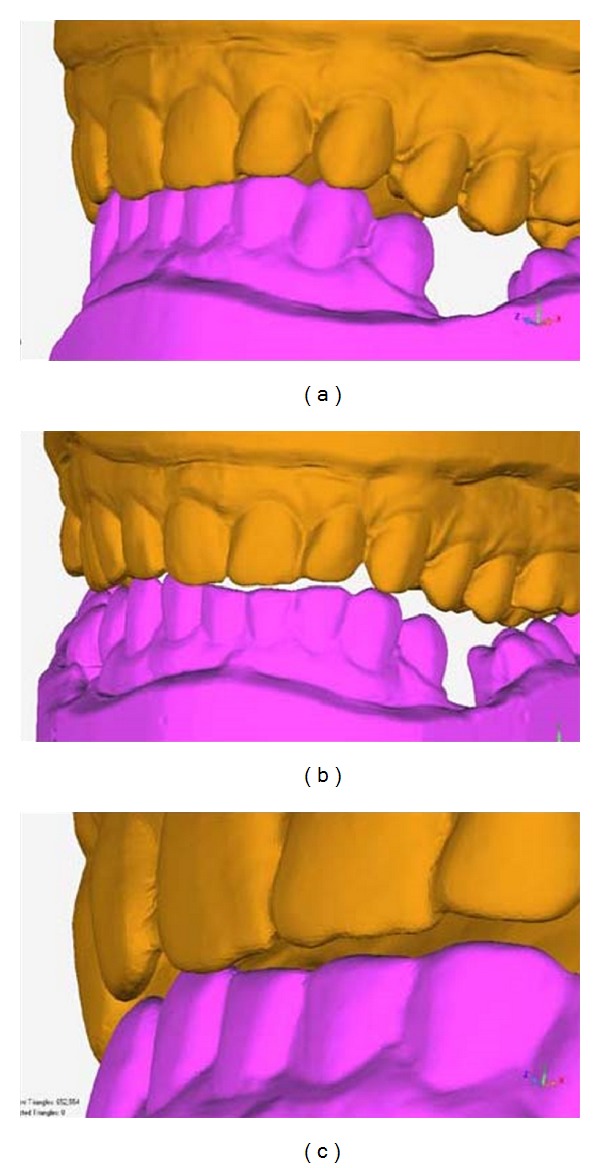
Three screen shots from a 4D animation of protrusion (a) shows the closed position, (b) an intermediate position, and (c) shows the single contact sliding on the right lateral incisors.

The use of USB 3 cameras is expected to provide about a 3× increase in speed-up to 25 frames/sec. This rate is sufficiently high not to require the interpolation of intermediate frames.  Figure 8 illustrates the motion that can be displayed, showing three screen shots from a protrusion sequence. 

## 8. Dynamic CAD

Recording protrusive and lateral excursive movements along with the clinical evaluation of the deflective movement of the guiding teeth could provide the information needed to develop the palatal contours of the teeth using CAD, so that the palatal surfaces would be physiologically in balance with the muscles of mastication and the neutral zone. 

The recording of the mandibular movement while chewing provides the information to CAD to develop the cusp heights and fossa contours of the posterior teeth to eliminate occlusal interferences, to provide physiologic muscle balance in the dentate patient, and to balance the denture occlusions for CAD/CAM dentures. 

Defining the opening and the closing of the mandible in relation to head posture would provide CAD/CAM with the information to provide the long centric related to the palatal surfaces of the maxillary anterior teeth to accommodate changes in head posture. 

## 9. Discussion

Dynamic CAD can provide dentistry with the technology to develop a true (patient-specific) virtual articulator to diagnose and restore a patient's occlusion within the physiologic parameters for that particular patient. Currently, occlusions are restored using either a mechanical articulator or a static CAD image, neither of which can provide the physiologic requirements of a patient's occlusion. 

The refinement of the imaging method described in this paper and the development of the 4D camera provide the initial technology to integrate a patient-specific motion into CAD/CAM technology. New design tools will be required to integrate the dynamic motion data into CAD. 

As development continues, increasingly larger surface areas are being imaged. Accurate full-arch animations seem to be within reach. Improved long-range registrations can also be achieved by combining scans taken on opposite sides of the mouth. 

Applications of this method include restorative, diagnostic, and surgical areas of dentistry. In addition to providing a virtual articulator, development of software to link dynamic CAD to CT or MRI may provide a diagnostic tool to develop a 3D image in motion that can be used to treat TMJ dysfunction. 

Commercially, the system would integrate with offices having all levels of technology. Offices with intraoral or table-top model scanners can transmit the digital 4D scans and model data to the laboratory. For offices with no technology, physical models would still have to be sent to the laboratory for scanning.

## 10. Conclusions

This work demonstrates the feasibility of dynamic CAD. The development of 4D technology and the true virtual articulator could have a profound effect in the future diagnosis and treatment of occlusal disease and TMJ disorders.
